# *BRAF*融合在非小细胞肺癌中的研究进展

**DOI:** 10.3779/j.issn.1009-3419.2023.101.28

**Published:** 2023-10-20

**Authors:** Ping XIAO, Diansheng ZHONG

**Affiliations:** 300052 天津，天津医科大学总医院肿瘤内科; Department of Medical Oncology, Tianjin Medical University General Hospital, Tianjin 300052, China

**Keywords:** 肺肿瘤, 鼠类肉瘤病毒癌基因同源物B1, 融合, Lung neoplasms, V-Raf murine sarcoma viral oncogene homolog B1, Fusion

## Abstract

在晚期非小细胞肺癌（non-small cell lung cancer, NSCLC）中，鼠类肉瘤病毒癌基因同源物B1（V-Raf murine sarcoma viral oncogene homolog B1, BRAF）突变恶性程度高、预后差，目前达拉非尼联合曲美替尼被批准用于BRAF V600突变患者的一线治疗。BRAF除了突变外，还可以发生融合，随着基因检测的发展，BRAF融合的检出逐渐增多，但是对于BRAF融合的治疗缺乏有效的治疗策略。本文从BRAF融合的临床特点、作用机制、临床治疗等方面进行综述，加强对BRAF基因融合的认识，为BRAF融合在NSCLC患者的治疗方面提供理论依据。

## Abstract (en)

In advanced non-small cell lung cancer (NSCLC), V-Raf murine sarcoma viral oncogene homolog B1 (BRAF) mutation is highly malignant and has poor prognosis, and currently Dabrafenib in combination with Trametinib is approved for first-line treatment of patients with BRAF V600 mutation. In addition to mutations, BRAF fusion can also occur. With the development of gene detection, the detection of BRAF fusion is gradually increasing, but there is a lack of effective therapeutic strategies for BRAF fusion. In this paper, we review the clinical characteristics, mechanism of action, and clinical treatment of BRAF fusion to provide a basis for the treatment of BRAF fusion in NSCLC patients.

肺癌是世界上发病率和死亡率居高不下的恶性肿瘤，其中非小细胞肺癌（non-small cell lung cancer, NSCLC）约占85%^[1]^。随着靶向药物、免疫检查点抑制剂（immune checkpoint inhibitors, ICIs）的飞速进步，肺癌的治疗格局发生了重大改变。与传统的化疗相比，靶向药物、ICIs治疗明显延长了无进展生存期（progression-free survival, PFS）和总生存期（overall survival, OS）。因此，寻求有效的靶点成为肺癌治疗的重要决策。

鼠类肉瘤病毒癌基因同源物B1（V-Raf murine sarcoma viral oncogene homolog B1, BRAF）在晚期NSCLC中的突变率为2%-6%^[2⇓-4]^。BRAF突变患者预后差，生存期较短，靶向药物的出现为这类患者带来了希望。目前指南推荐达拉非尼联合曲美替尼作为BRAF V600突变的晚期或转移性NSCLC患者的首选治疗^[5,6]^。

BRAF除了突变之外，还可以发生断裂重排，形成新的融合基因。BRAF融合最早在2005年在甲状腺癌中被报道^[7]^，开启了BRAF融合在肿瘤治疗的探索。目前研究最多的是毛细胞星形细胞瘤，大约60%的毛细胞星形细胞瘤会出现BRAF融合^[8]^。随着基因检测的应用，尤其是第二代测序技术的深入，越来越多BRAF融合在其他癌种中被发现。2017年斯隆-凯特林癌症研究所（Memorial Sloan-Kettering Cancer Center, MSKCC）应用MSK-IMPACT检测方法，对10,000例晚期实体瘤患者进行第二代测序，发现BRAF融合39例，其中NSCLC检出率为0.38%，结肠癌为0.2%，乳腺癌为0.08%，胰腺癌为1.43%，前列腺癌为0.8%，胆囊癌为0.41%，卵巢癌为0.46%，胶质瘤为0.78%，生殖细胞肿瘤为0.37%，软组织肿瘤为0.46%^[9]^。融合基因可能是致癌的驱动因素，也是靶向治疗的潜在目标。在NSCLC患者中，尤其表皮生长因子受体（epidermal growth factor receptor, EGFR）靶向药物耐药后的患者，BRAF融合的检出逐渐增多，然而BRAF融合的治疗仍参照非驱动基因突变的患者，缺乏有效的治疗策略，因此加强对BRAF融合的认识并探索更加有针对性的治疗方案势在必行。本文就BRAF融合在NSCLC患者中的临床特点、作用机制、临床治疗等方面进行综述，为BRAF融合的治疗提供更多的选择。

## 1 BRAF基因的生物学特征

BRAF是一种原癌基因，定位于7号染色体，包含18个外显子，编码BRAF蛋白。从N端到C端依次是高度保守区（conserved regions, CR）1、CR2和CR3三个CR，其中CR1区由RAS蛋白结合区和富含半胱氨酸区组成，可以与RAS-三磷酸鸟苷（guanosine triphosphate, GTP）结合；CR2区富含丝氨酸/苏氨酸，调节磷酸化RAF激酶活性；CR3区为三磷酸腺苷（adenosine triphosphate, ATP）结合位点和激活区，含有酪氨酸和丝氨酸残基及有多个磷酸化位点，磷酸化后可激活BRAF蛋白和诱导性激活细胞外信号调节激酶（extracellular signal-regulated kinase, ERK）^[10]^。

BRAF作为RAS-RAF-MEK-ERK通路的上游调节因子，参与丝裂原活化蛋白激酶（mitogen activated protein kinase, MAPK）通路的活化。当细胞外生长因子与细胞表面的受体结合后，活化膜结合蛋白RAS，活化的RAS蛋白使BRAF二聚化，并磷酸化激活MAPK激酶（MAPK kinase, MEK）进一步激活ERK，促进肿瘤细胞的增殖、分化、迁移。

依据是否具有激酶活性、对RAS活化的依赖性和RAF的二聚化特征，BRAF突变可分为I、II、III类^[11]^。其中，I类突变为具有高激酶活性单体突变，不依赖RAS活性，且不需要二聚体来激活下游ERK通路，激活的ERK对RAS蛋白产生负调控，导致RAS活性降低。I类突变主要发生于CR3（BRAF激酶结构域）的外显子11和15，以BRAF V600突变为代表。II类突变为具有激酶活性的二聚体且不依赖RAS活化。这类突变使活化区与p-LOOP交互作用解除，解除激酶的自身抑制状态。III类突变依赖RAS活化，通过野生型CRAF的二聚作用，激活信号，为无激酶活性的异源二聚体，具有高RAS-GTP活化水平^[12]^。

## 2 BRAF融合NSCLC临床特点

在一项大样本NSCLC患者研究^[13]^中，应用Foundation Medicine CGP检测17,128例患者的石蜡标本，发现42例BRAF融合，BRAF融合仅占0.2%，非常罕见。BRAF融合主要发生在肺腺癌患者中，中位年龄为67岁（范围：44-93岁），55%为女性患者，48%在原发灶检出，52%在转移灶检出。与BRAF融合基因共存的常见驱动基因包括TP53（67%）、CDKN2A（31%）、EGFR（29%）和CDKN2B（26%）^[13]^。BRAF融合患者的肿瘤突变负荷（tumor mutational burden, TMB）较低，中位数为3.8 mut/Mb，其中7%（3/42）的患者TMB>20 mut/Mb，35%（15/42）的患者TMB在6.1-19.9 mut/Mb，57%（24/42）的患者TMB在0-6 mut/Mb^[13]^。

在EGFR突变患者中，应用EGFR-酪氨酸激酶抑制剂（tyrosine kinase inhibitors, TKIs）耐药后，BRAF融合检出率为1%-2%^[14⇓-16]^。BRAF融合的出现导致细胞MEK、ERK通路的磷酸化增加，下游MAPK信号通路组成性激活，进而影响肿瘤细胞对抗EGFR靶向治疗的敏感性，揭示BRAF融合为EGFR-TKIs获得性耐药的一种机制。Peng等^[17]^在中国EFGR突变人群中，发现EGFR-TKIs治疗耐药后合并BRAF融合的17例患者中有12例为EGFR 19del，提示BRAF融合在EGFR 19del的患者中更易出现（P=0.015）。此外，在BRAF V600E突变的黑色素瘤中，BRAF抑制剂联合MEK抑制剂治疗后，检出BRAF融合基因，表明BRAF融合可能为BRAF抑制剂获得性耐药的一种机制^[18]^。

## 3 BRAF融合作用机制

BRAF I类突变导致MAPK持续活化，负反馈抑制RAS，阻止BRAF二聚化，保持单体构象。与导致激酶结构域突变的BRAF单体突变相反，BRAF融合蛋白保留正常的激酶结构域，BRAF基因融合导致N端CR1自动抑制区域断裂，无法有效抑制BRAF活化，导致BRAF基因形成二聚体而结构性持续活化，且不依赖上游RAS活性，功能类似于II类突变^[15,19]^。活化后的BRAF导致下游MAPK信号通路异常激活。BRAF融合蛋白表达未突变的激酶结构域，可能表现出多种构象状态，对小分子抑制剂（如Vemurafenib）的亲和力降低^[20]^。

## 4 BRAF融合伴侣

目前肺癌中发现众多BRAF融合伴侣，包括AGAP3、AGK、ARMC10、SALL2、DOCK4、EPS515、GHR、KIAA1549、TRIM24、AP3B1、KCND2、GRM8、PARP12、BIN1、EYS、TRIO、PTPRN2、LMO7、PTPN13、UBN2、ZC3HAV1、ERC1、BTFL34、EPS15、MKRN1、NUP214、STAT3、ZC3HAV4等^[13,21⇓-23]^，63%的融合体与BRAF位于同一条7号染色体上^[24]^，其中最常见的融合体为AGK、DOCK4和TRIM24。BRAF融合断点常位于已知的热点区域（外显子7-11）^[17]^，保留了BRAF二聚化基序和BRAF激活区。融合基因的表达依赖于融合伴侣启动子，融合伴侣通常具有二聚化或寡聚化序列，5’端的伴侣编码卷曲螺旋结构域，使激酶二聚化。然而BRAF具有自己的二聚化基序，跨越外显子12和13，在目前已知的BRAF融合体中BRAF二聚化基序是完整的，因此，BRAF融合功能可能不需要具有二聚化能力的5’伴侣^[25]^。

## 5 BRAF融合的治疗

对于BRAF融合，目前指南没有相关针对抗BRAF治疗的相关推荐。不过，其他癌症、临床前研究及个案报道对BRAF融合的治疗会有所启示（表1）。

**表1:** BRAF融合肺癌病例汇总

Case	Histologic diagnosis	Age (yr)/ gender	BRAF fusion	Primary/ secondary	Therapy	Line of therapy	PFS (mon)	Response	OS (mon)	Adverse events
Kong WM^[42]^	Lung adenocarcinoma	53/F	BTN2A1-BRAF	EGFR 19 resistance	Duvalizumab	5	<3	PD	<3	NA
Kian W^[38]^	Lung adenocarcinoma	75/F	MKRN1-BRAF	EGFR 19 resistance	Trametinib and Dabrafenib and Osimertinib	4	NA	PR	NA	NA
Dagogo-Jack I^[45]^	Lung adenocarcinoma	59/F	AGK-BRAF	EGFR 19 resistance	Trametinib and Osimertinib	5	5	PR	NA	2 diarrhea, grade 2 fatigue, grade 1 rash, and grade 1 gastrointestinal bleed
Chou YT^[37]^	Lung poorly differentiated adenocarcinoma	67/M	KIAA1549- BRAF and MET amplification	NA	Dabrafenib, Trametinib and Capmatinib	5	>6	SD	NA	3 peripheral edema, 1 nausea, 2 fatigue, 1 skin rash and 1 fever
Yu Y^[29]^	Lung adenocarcinoma	60/M	SND1-BRAF Fusion	Primary	Trametinib	5	>4	PR	NA	1 scattered acne with itching over the head and face
Luo J^[23]^	Lung adenocarcimoma	49/M	ZC3HAV1-BRAF fusion	EGFR 21 resistance	Erlotinib and Trametinib	3	>5.5	PR	NA	NA
Luo J^[23]^	Lung adenocarcinoma	60/F	ERC1-BRAF fusion	EGFR-sensitizing mutation resistance	Erlotinib and Trametinib	4	1.7	PD	NA	NA
Wang CY^[28]^	Lung adenocarcinoma	66/M	LIMD1-BRAF fusion	Primary	Trametinib	1	7.4	PR	NA	1 scattered acne

BRAF: V-Raf murine sarcoma viral oncogene homolog B1; NSCLC: non-small cell lung cancer; F: female; M: male; EGFR: epidermal growth factor receptor; PFS: progression-free survival; OS: overall survival; PR: partial response; SD: stable disease; PD: progressive disease; NA: not available; TPS: tumor proportion score.

### 5.1 BRAF融合与靶向治疗

#### 5.1.1 BRAF抑制剂单药

第一代RAF抑制剂（包括达拉菲尼和威罗菲尼）针对激活的BRAF蛋白，与ATP竞争结合位点，导致活性位点无法暴露。第一代RAF抑制剂优先抑制BRAF单体，因其与BRAF二聚体中的一个位点结合，显著降低其与第二个位点的亲和力，因此对具有激酶活性的二聚体是无效的。在BRAF融合细胞系中，应用第一代BRAF抑制剂后，导致BRAF融合蛋白的反常激活以及MEK磷酸化增加^[26]^，无法有效抑制肿瘤生长。多项临床前研究^[15,18,27]^显示第一代BRAF抑制剂不能克服由于BRAF融合导致的耐药。而正在临床研究阶段的新一代RAF抑制剂-RAF二聚体阻断剂（PLX8394、FORE8394等）以及泛-RAF（pan-RAF）抑制剂，通过抑制前聚体，避免BRAF反常激活，不仅可有效抑制BRAF V600单体突变，同时能对BRAF II/III类突变以及BRAF融合有效^[26]^，这类药物未来可能对BRAF融合突变的治疗带来新的改变。

#### 5.1.2 MEK抑制剂单药

曲美替尼是MEK1和MEK2激酶的可逆性抑制剂，通过对MEK蛋白的抑制作用，影响MAPK信号通路，抑制细胞增殖。对于BRAF V600突变的患者，曲美替尼联合BRAF抑制剂具有协同抗肿瘤的作用。在BRAF融合的患者中，多项临床前研究及个案报道发现曲美替尼对BRAF融合有效。Wang等^[28]^报告1例原发LIMD1-BRAF融合的肺腺癌，患者拒绝化疗，一线应用曲美替尼抗肿瘤治疗，PFS达7.4个月。另外1例晚期肺腺癌，初始检测出SND1-BRAF融合，经过化疗、抗血管、免疫等多线治疗后，检测出SND1-BRAF以及BRAF-RNF150融合，给予曲美替尼治疗后，症状明显缓解，病情评估为部分缓解^[29]^。在其他癌症中，如黑色素瘤^[18]^、前列腺癌^[30]^、胶质瘤^[31]^等，曲美替尼对于BRAF融合的患者治疗有效也有报道。因此，对于BRAF融合的患者可考虑采用曲美替尼进行治疗。

#### 5.1.3 多激酶抑制剂

多激酶抑制剂包括索拉菲尼、瑞戈非尼、培唑帕尼等，这类药物为非特异性BRAF抑制剂，与BRAF非激酶活性构象相结合，针对的是未活化的BRAF蛋白，通过结合到BRAF蛋白的“口袋”上，导致ATP分子无法进入，抑制肿瘤细胞的生长，对包括BRAF在内的多种激酶均有抑制作用。在黑色素瘤、前列腺癌细胞系中，索拉菲尼可以抑制BRAF融合细胞生长^[32,33]^。不过这类药物单用抗肿瘤作用有限，在BRAF融合的恶性黑色素细胞瘤和恶性梭形细胞瘤中，有索拉菲尼联合治疗有效的个案报告^[34,35]^。

#### 5.1.4 RAF抑制剂联合MEK抑制剂

目前在BRAF融合黑色素瘤异种模型中，pan-RAF抑制剂联合MEK抑制剂具有协同抗肿瘤的作用^[24]^。对于奥希替尼耐药的患者，临床前研究^[36]^表明，EGFR-MEK途径的双重抑制优于单独抑制MEK信号通路。在个案报道中，BRAF抑制剂联合MEK抑制剂对于BRAF融合的患者疗效显著。Chou等^[37]^报告1例分化差的晚期肺腺癌患者，一线接受化疗联合帕博丽珠单抗，疾病进展后基因检测同时检出KIAA1549-BRAF融合和MET扩增，后续给予卡博替尼、达拉非尼和曲美替尼治疗有效，疾病控制超过6个月。另外1例EGFR 19del患者，一线厄洛替尼治疗耐药后，检出T790M突变，二线奥希替尼治疗，耐药后检出MKRN1-BRAF融合，三线化疗联合奥希替尼治疗疾病进展，四线予奥希替尼联合曲美替尼、达拉非尼，疾病得到控制^[38]^，但是需要关注联合治疗导致的不良反应。

#### 5.1.5 MEK/PI3K抑制剂或MEK/CDK4/6抑制剂联合

在恶性肿瘤中，大多会出现MAPK、细胞周期以及磷脂酰肌醇3-激酶-哺乳动物雷帕霉素靶蛋白（phosphatidylinositol 3-kinase-mammalian target of rapamycin, PI3K-mTOR）信号通路改变，联合抑制这些信号通路可能对抗肿瘤起到协同作用。在黑色素瘤BRAF融合细胞系中，与单用MEK抑制剂相比，应用MEK抑制剂联合PI3K抑制剂或MEK抑制剂联合CDK4/6抑制剂具有协同抗肿瘤作用^[39]^。这些联合治疗也是未来探索的一个方向。

### 5.2 BRAF融合与免疫治疗

驱动基因阳性的患者，ICIs的疗效差强人意。对于BRAF突变的患者具有较高的TMB和程序性死亡配体1（programmed cell death ligand 1, PD-L1）表达水平，可潜在从ICIs治疗中获益。研究^[40]^发现BRAF突变的患者单药ICIs的客观缓解率（objective response rate, ORR）为10%-30%，中位PFS为2-4个月，与驱动基因野生型NSCLC二线治疗中的疗效类似，并且对于BRAF非600E突变的患者，免疫治疗的疗效更好一些。不过对于功能类似于II类突变BRAF融合的患者，总体TMB较低（中位数为3.8 mut/Mb）^[13]^，免疫获益可能性较小。在黑色素细胞瘤中，Panning等^[41]^报告5例BRAF融合/重排的患者，应用ICIs治疗后，4例出现疾病进展，提示免疫治疗疗效不佳。在NSCLC中，BRAF融合免疫治疗效果尚不明确，目前仅在个案报道中有少量报道。Yu等^[29]^报告1例初始存在BRAF融合合并PD-L1（应用22C3抗体）肿瘤细胞阳性比例分数（tumor proportion score, TPS）为30%的肺腺癌患者，一线化疗联合抗血管进展后，免疫单药以及联合化疗治疗效果不佳，后续给予曲美替尼治疗后达部分缓解。另外Kong等^[42]^报告1例肺腺癌患者接受EGFR靶向药物耐药后，检出BTN2A1-BRAF融合，并且PD-L1高表达（TPS为90%，应用22C3抗体），然而给予度伐利尤单抗单药治疗无效。

在结肠癌中，BRAF V600E突变与CpG岛甲基化表型相关，导致MLH1启动子过度甲基化，微卫星高度不稳定。而基因融合同样可导致MAPK信号通路活化，MLH1基因启动子CpG岛发生甲基化，使得细胞发生微卫星不稳定，患者可能从免疫治疗获益^[43]^。在一项回顾性研究^[43]^中，2314例结肠癌患者发现21例存在基因融合（包括8例NTRK、2例FGFR、1例ROS1、1例ALK、4例RET、5例BRAF等），其中57%（12/21）为微卫星高度不稳定状态，并且这些患者均存在MLH1表达缺失。Heinrich等^[44]^报告在1例胰腺癌患者，存在TRIM24-BRAF融合，同时患者存在MLH1启动子超甲基化，微卫星高度不稳定状态，一线给予帕博丽珠单抗治疗后，肿瘤达到持久的控制。因此，对于BRAF融合的患者，可以考虑行DNA错配修复系统/微卫星不稳定状态检测。

## 6 未来挑战与方向

BRAF融合的患者比例低，治疗手段有限，很难开展前瞻性临床研究。目前其治疗策略仍类似于驱动基因阴性的患者。基于检测方法的局限性、检测标本的差异以及实验室条件的不同，很可能低估了激酶融合导致的肿瘤患者人数。未来可以考虑因地制宜选择合适的检测方式，以最大限度地为患者提供精准治疗。基于以上临床前研究以及个案报道，对于BRAF融合的患者仍需要进行更多的探索。免疫治疗差强人意，MEK抑制剂、新型RAF抑制剂以及联合治疗模式显示出明显的抗肿瘤效果，未来需要进一步进行多中心的临床研究或“篮子”试验，为BRAF融合治疗提供更可靠的理论依据。此外，在ALK基因融合中，不同的融合位点及融合方式对靶向药物的敏感性及耐药方式不同，但是关于BRAF不同的融合伴侣是否影响药物的敏感性和继发性耐药目前尚不清楚，以及不同癌种对治疗的敏感性是否存在差异，这也是未来需要进一步进行探索的方向。因此，BRAF融合有望成为NSCLC潜在的治疗靶点，为肺癌的个体化治疗提供新的方向。

## References

[1] Sung H, Ferlay J, Siegel RL, et al. Global cancer statistics 2020: GLOBOCAN estimates of incidence and mortality worldwide for 36 cancers in 185 countries. CA Cancer J Clin, 2021, 71(3): 209-249. doi: 10.3322/caac.21660 33538338

[2] Barlesi F, Mazieres J, Merlio JP, et al. Routine molecular profiling of patients with advanced non-small-cell lung cancer: results of a 1-year nationwide programme of the French Cooperative Thoracic Intergroup (IFCT). Lancet, 2016, 387(10026): 1415-1426. doi: 10.1016/S0140-6736(16)00004-0 26777916

[3] Villaruz LC, Socinski MA, Abberbock S, et al. Clinicopathologic features and outcomes of patients with lung adenocarcinomas harboring BRAF mutations in the Lung Cancer Mutation Consortium. Cancer, 2015, 121(3): 448-456. doi: 10.1002/cncr.29042 25273224 PMC4305000

[4] Kris MG, Johnson BE, Berry LD, et al. Using multiplexed assays of oncogenic drivers in lung cancers to select targeted drugs. JAMA, 2014, 311(19): 1998-2006. doi: 10.1001/jama.2014.3741 24846037 PMC4163053

[5] Ettinger DS, Wood DE, Aisner DL, et al. NCCN guidelines® insights: non-small cell lung cancer, version 2. 2023. J Natl Compr Canc Netw, 2023, 21(4): 340-350. doi: 10.6004/jnccn.2023.0020 37015337

[6] Planchard D, Popat S, Kerr K, et al. Metastatic non-small cell lung cancer: ESMO Clinical Practice Guidelines for diagnosis, treatment and follow-up. Ann Oncol, 2018, 29(Suppl 4): iv192-iv237. doi: 10.1093/annonc/mdy275 30285222

[7] Ciampi R, Knauf JA, Kerler R, et al. Oncogenic AKAP9-BRAF fusion is a novel mechanism of MAPK pathway activation in thyroid cancer. J Clin Invest, 2005, 115(1): 94-101. doi: 10.1172/JCI23237 15630448 PMC539203

[8] Jones DT, Kocialkowski S, Liu L, et al. Tandem duplication producing a novel oncogenic BRAF fusion gene defines the majority of pilocytic astrocytomas. Cancer Res, 2008, 68(21): 8673-8677. doi: 10.1158/0008-5472.CAN-08-2097 18974108 PMC2577184

[9] Zehir A, Benayed R, Shah RH, et al. Mutational landscape of metastatic cancer revealed from prospective clinical sequencing of 10,000 patients. Nat Med, 2017, 23(6): 703-713. doi: 10.1038/nm.4333 28481359 PMC5461196

[10] Pollock PM, Meltzer PS. A genome-based strategy uncovers frequent BRAF mutations in melanoma. Cancer Cell, 2002, 2(1): 5-7. doi: 10.1016/s1535-6108(02)00089-2 12150818

[11] Frisone D, Friedlaender A, Malapelle U, et al. A BRAF new world. Crit Rev Oncol Hematol, 2020, 152: 103008. doi: 10.1016/j.critrevonc.2020.103008 32485528

[12] Lin Q, Zhang H, Ding H, et al. The association between BRAF mutation class and clinical features in BRAF-mutant Chinese non-small cell lung cancer patients. J Transl Med, 2019, 17(1): 298. doi: 10.1186/s12967-019-2036-7 31470866 PMC6716889

[13] Reddy VP, Gay LM, Elvin JA, et al. BRAF fusions in clinically advanced non-small cell lung cancer: An emerging target for anti-BRAF therapies. J Clin Oncol, 2017, 35(15_suppl): 9072. doi: 10.1200/JCO.2017.35.15_suppl.9072

[14] Yu HA, Suzawa K, Jordan E, et al. Concurrent alterations in EGFR-mutant lung cancers associated with resistance to EGFR kinase inhibitors and characterization of MTOR as a mediator of resistance. Clin Cancer Res, 2018, 24(13): 3108-3118. doi: 10.1158/1078-0432.CCR-17-2961 29530932 PMC6420806

[15] Vojnic M, Kubota D, Kurzatkowski C, et al. Acquired BRAF rearrangements induce secondary resistance to EGFR therapy in EGFR-mutated lung cancers. J Thorac Oncol, 2019, 14(5): 802-815. doi: 10.1016/j.jtho.2018.12.038 30831205 PMC6486868

[16] Leonetti A, Sharma S, Minari R, et al. Resistance mechanisms to osimertinib in EGFR-mutated non-small cell lung cancer. Br J Cancer, 2019, 121(9): 725-737. doi: 10.1038/s41416-019-0573-8 31564718 PMC6889286

[17] Peng P, Lv G, Hu J, et al. Co-mutations of epidermal growth factor receptor and BRAF in Chinese non-small cell lung cancer patients. Ann Transl Med, 2021, 9(16): 1321. doi: 10.21037/atm-21-3570 34532458 PMC8422152

[18] Kulkarni A, Al-Hraishawi H, Simhadri S, et al. BRAF fusion as a novel mechanism of acquired resistance to vemurafenib in BRAF V600E mutant melanoma. Clin Cancer Res, 2017, 23(18): 5631-5638. doi: 10.1158/1078-0432.CCR-16-0758 28539463

[19] Rosell R, González-Cao M, Codony-Servat J, et al. Acquired BRAF gene fusions in Osimertinib resistant EGFR-mutant non-small cell lung cancer. Transl Cancer Res, 2023, 12(3): 456-460. doi: 10.21037/tcr-22-2888 37033354 PMC10080327

[20] Karoulia Z, Gavathiotis E, Poulikakos PI. New perspectives for targeting RAF kinase in human cancer. Nat Rev Cancer, 2017, 17: 676-691. doi: 10.1038/nrc.2017.79 28984291 PMC6000833

[21] Schrock AB, Zhu VW, Hsieh WS, et al. Receptor tyrosine kinase fusions and BRAF kinase fusions are rare but actionable resistance mechanisms to EGFR tyrosine kinase inhibitors. J Thorac Oncol, 2018, 13(9): 1312-1323. doi: 10.1016/j.jtho.2018.05.027 29883838

[22] Sheikine Y, Pavlick D, Klempner SJ, et al. BRAF in lung cancers: analysis of patient cases reveals recurrent BRAF mutations, fusions, kinase duplications, and concurrent alterations. JCO Precis Oncol, 2018, 2: PO. 17.00172. doi: 10.1200/PO.17.00172 PMC7446447 32913992

[23] Luo J, Makhnin A, Tobi Y, et al. Erlotinib and trametinib in patients with EGFR-mutant lung adenocarcinoma and acquired resistance to a prior tyrosine kinase inhibitor. JCO Precis Oncol, 2021, 5: PO. 20.00315. doi: 10.1200/PO.20.00315 PMC8232136 34250388

[24] Botton T, Talevich E, Mishra VK, et al. Genetic heterogeneity of BRAF fusion kinases in melanoma affects drug responses. Cell Rep, 2019, 29(3): 573-588.e7. doi: 10.1016/j.celrep.2019.09.009 31618628 PMC6939448

[25] Baljuls A, Mahr R, Schwarzenau I, et al. Single substitution within the RKTR motif impairs kinase activity but promotes dimerization of RAF kinase. J Biol Chem, 2011, 286(18): 16491-16503. doi: 10.1074/jbc.M110.194167 21454547 PMC3091254

[26] Sievert AJ, Lang SS, Boucher KL, et al. Paradoxical activation and RAF inhibitor resistance of BRAF protein kinase fusions characterizing pediatric astrocytomas. Proc Natl Acad Sci USA, 2013, 110(15): 5957-5962. doi: 10.1073/pnas.1219232110 23533272 PMC3625308

[27] Piotrowska Z, Isozaki H, Lennerz JK, et al. Landscape of acquired resistance to osimertinib in EGFR-mutant NSCLC and clinical validation of combined EGFR and RET inhibition with osimertinib and BLU-667 for acquired RET fusion. Cancer Discov, 2018, 8(12): 1529-1539. doi: 10.1158/2159-8290.CD-18-1022 30257958 PMC6279502

[28] Wang CY, Hsia JY, Li CH, et al. Lung adenocarcinoma with primary LIMD1-BRAF fusion treated with MEK inhibitor: a case report. Clin Lung Cancer, 2021, 22(6): e878-e880. doi: 10.1016/j.cllc.2021.05.003 34148767

[29] Yu Y, Yu M, Li Y, et al. Rapid response to monotherapy with MEK inhibitor trametinib for a lung adenocarcinoma patient harboring primary SDN1-BRAF fusion: A case report and literature review. Front Oncol, 2022, 12: 945620. doi: 10.3389/fonc.2022.945620 36059688 PMC9437588

[30] Fenor MD, Ruiz-Llorente S, Rodríguez-Moreno JF, et al. MEK inhibitor sensitivity in BRAF fusion-driven prostate cancer. Clin Transl Oncol, 2022, 24(12): 2432-2440. doi: 10.1007/s12094-022-02916-6 35994225

[31] Wagner LM, Myseros JS, Lukins DE, et al. Targeted therapy for infants with diencephalic syndrome: A case report and review of management strategies. Pediatr Blood Cancer, 2018, 65(5): e26917. doi: 10.1002/pbc.26917 29369501

[32] Botton T, Yeh I, Nelson T, et al. Recurrent BRAF kinase fusions in melanocytic tumors offer an opportunity for targeted therapy. Pigment Cell Melanoma Res, 2013, 26(6): 845-851. doi: 10.1111/pcmr.12148 23890088 PMC3808507

[33] Palanisamy N, Ateeq B, Kalyana-Sundaram S, et al. Rearrangements of the RAF kinase pathway in prostate cancer, gastric cancer and melanoma. Nat Med, 2010, 16(7): 793-798. doi: 10.1038/nm.2166 20526349 PMC2903732

[34] Subbiah V, Westin SN, Wang K, et al. Targeted therapy by combined inhibition of the RAF and mTOR kinases in malignant spindle cell neoplasm harboring the KIAA1549-BRAF fusion protein. J Hematol Oncol, 2014, 7: 8. doi: 10.1186/1756-8722-7-8 24422672 PMC3896681

[35] Passeron T, Lacour JP, Allegra M, et al. Signalling and chemosensitivity assays in melanoma: is mutated status a prerequisite for targeted therapy?. Exp Dermatol, 2011, 20(12): 1030-1032. doi: 10.1111/j.1600-0625.2011.01385.x 22092579

[36] Dummer R, Ascierto PA, Gogas HJ, et al. Encorafenib plus binimetinib versus vemurafenib or encorafenib in patients with BRAF-mutant melanoma (COLUMBUS): a multicentre, open-label, randomised phase 3 trial. Lancet Oncol, 2018, 19(5): 603-615. doi: 10.1016/S1470-2045(18)30142-6 29573941

[37] Chou YT, Lin CC, Lee CT, et al. Durable response of dabrafenib, trametinib, and capmatinib in an NSCLC patient with co-existing BRAF-KIAA 1549 fusion and MET amplification: a case report. Front Oncol, 2022, 12: 838798. doi: 10.3389/fonc.2022.838798 35372088 PMC8972191

[38] Kian W, Krayim B, Alsana H, et al. Overcoming CEP85L-ROS1, MKRN1-BRAF and MET amplification as rare, acquired resistance mutations to Osimertinib. Front Oncol, 2023, 13: 1124949. doi: 10.3389/fonc.2023.1124949 36923435 PMC10009227

[39] Kim HS, Jung M, Kang HN, et al. Oncogenic BRAF fusions in mucosal melanomas activate the MAPK pathway and are sensitive to MEK/PI3K inhibition or MEK/CDK4/6 inhibition. Oncogene, 2017, 36(23): 3334-3345. doi: 10.1038/onc.2016.486 28092667

[40] Yan N, Guo S, Zhang H, et al. BRAF-mutated non-small cell lung cancer: current treatment status and future perspective. Front Oncol, 2022, 12: 863043. doi: 10.3389/fonc.2022.863043 35433454 PMC9008712

[41] Panning A, Samlowski W, Allred G. Lack of influence of non-overlapping mutations in BRAF, NRAS, or NF1 on 12-month best objective response and long-term survival after checkpoint inhibitor-based treatment for metastatic melanoma. Cancers (Basel), 2023, 15(13): 3527. doi: 10.3390/cancers15133527 PMC10340344 37444637

[42] Kong WM, Guo YJ, Ma J, et al. BTN2A1-BRAF fusion may be a novel mechanism of resistance to osimertinib in lung adenocarcinoma: a case report. Transl Cancer Res, 2023, 12(1): 186-193. doi: 10.21037/tcr-22-2060 36760378 PMC9906054

[43] Cocco E, Benhamida J, Middha S, et al. Colorectal carcinomas containing hypermethylated MLH 1 promoter and wild-type BRAF/KRAS are enriched for targetable kinase fusions. Cancer Res, 2019, 79(6): 1047-1053. doi: 10.1158/0008-5472.CAN-18-3126 30643016 PMC6420871

[44] Heinrich K, Fischer LE, De Toni EN, et al. Case of a patient with pancreatic cancer with sporadic microsatellite instability associated with a BRAF fusion achieving excellent response to immunotherapy. JCO Precis Oncol, 2023, 7: e2200650. doi: 10.1200/PO.22.00650 37364232 PMC10309529

[45] Dagogo-Jack I, Piotrowska Z, Cobb R, et al. Response to the combination of osimertinib and trametinib in a patient with EGFR-mutant NSCLC harboring an acquired BRAF fusion. J Thorac Oncol, 2019, 14(10): e226-e228. doi: 10.1016/j.jtho.2019.05.046 31558234

